# Influence of uncemented humeral stem proximal geometry on stress distributions and torsional stability following total shoulder arthroplasty

**DOI:** 10.1186/s40634-019-0178-4

**Published:** 2019-02-25

**Authors:** Johannes Barth, Jérôme Garret, Laurent Geais, Hugo Bothorel, Mo Saffarini, Shoulder Friends Institute, Arnaud Godenèche

**Affiliations:** 1 Department of Orthopaedic Surgery, Centre Osteoarticulaire des Cèdres, Grenoble, France; 2 Clinique du Parc, Lyon, France; 3 Move-Up SAS, Alixan, France; 4 ReSurg SA, Chemin de la Vuarpillière 35, 1260 Nyon, Switzerland; 5 Shoulder Friends Institute, Paris, France; 6 Ramsay Générale de Santé, Hôpital Privé Jean Mermoz, Centre Orthopédique Santy, Lyon, France

**Keywords:** Shoulder arthroplasty, Proximal humerus, Implant design, FEA, Stress shielding, Torsional stability

## Abstract

**Background:**

While surgeons tend to implant larger stems to improve torsional stability, numerous studies demonstrated that increasing humeral stem diameter could exacerbate stress-shielding and lead to bone resorption. We aimed to determine the influence of humeral stem proximal geometry on stress distributions and torsional stability following total shoulder arthroplasty.

**Methods:**

Preoperative computed tomography scans were acquired from 5 patients and processed to form 3-dimensional models of the proximal humerus. Computer models of 3 generic implants were created based on three designs: predominantly oval, semi-angular, and predominantly angular. All stems shared identical head geometry and differed only in the proximal metaphyseal area. Finite element analyses were performed, with the humerus rigidly constrained distally, and loaded to simulate the joint reaction force. Implant torsional stability and proximal bone stress distributions were assessed for the three different stem designs with three sizes: oversized (stem making contact with the cortical diaphysis), normosized (one increment smaller) and undersized (two increments smaller).

**Results:**

Considering the normosized stems, the angular design *increased* the physiologic bone stresses at the proximal section by 39–42%, while the oval and semi-angular designs *reduced* them by 5–9% and 8–13%, respectively. The oval design exhibited a median rotation of 2.1°, while the semi-angular and angular designs exhibited median rotations of 1.8°.

**Conclusion:**

The semi-angular stem granted an adequate compromise between physiologic stress distributed by the oval stem and torsional stability of the angular stem. Surgeons should be aware of the various benefits and drawbacks of the different humeral stem designs to ensure adequate torsional stability and physiologic loading.

## Background

The success of total shoulder arthroplasty (TSA) depends largely on sizing and positioning of the humeral and glenoid components, which can be challenging to optimize relying solely on intra-operative assessment. While the most frequent cause of complications and revisions after TSA is failure of the glenoid component (Pomwenger et al., 2014), loosening or periprosthetic fractures are sometimes also observed around the humeral stem (Quental et al., 2012; Verborgt et al., 2007).

Nagels et al. (2003) demonstrated that stem size directly influences the extents and zones of stress-shielding in the proximal humerus, which could therefore cause peripheral bone resorption (Quental et al., 2012; Razfar et al., 2016). Recent studies evaluated that signs of stress-shielding in the proximal humerus can be found in 40% to 60% of uncemented short stems at a follow-up of 7–8 years (Raiss et al., 2014; Schnetzke et al., 2018). Notably, unlike the weight-bearing hip joint, muscles surrounding the shoulder joint transmit loads more proximally (Quental et al., 2012), rendering the humeral metaphysis more sensitive to stimulus than the femoral metaphysis. In the last decade, implant manufacturers introduced stemless humeral heads as well as shortened humeral stems to reduce these complications and facilitate revision if required (Quental et al., 2012; Razfar et al., 2016). Recently, Razfar et al. (2016) evaluated, using finite element analysis (FEA), the influence of stem length on bone stresses and demonstrated that shorter stems could reduce stress-shielding in the proximal humerus (Razfar et al., 2016). It is therefore essential to optimize humeral implant size and proximal geometry to limit stress-shielding and ensure adequate implant stability and osteointegration.

Several authors evaluated the mechanical behavior of glenoid implants within the scapula (Buchler & Farron, 2004; Chevalier et al., 2016; Iannotti et al., 2005; Pomwenger et al., 2014; Stone et al., 1999), but very few studied the stress distribution or bone remodeling around the stem on the humeral side (Quental et al., 2012; Razfar et al., 2016). The purpose of this study was therefore to determine, using FEA, the influence of humeral stem size and proximal geometry on stress distributions and torsional stability within periprosthetic bone.

## Methods

The authors analyzed computed tomography (CT) shoulder scans of patients scheduled to receive TSA, and selected 5 shoulders that represent a wide spectrum of size, sex and age, known to denote bone quality (Kirchhoff et al., 2012). The selection comprised 2 women (aged 82 and 87; head diameters 55 and 47 mm) and 3 men (aged 56, 59, and 73; head diameter 63, 49, and 59 mm). The number of subjects included in this study was comparable to those of other FEA studies investigating stress distribution within the humerus (Chevalier et al., 2016; Quental et al., 2012; Razfar et al., 2016). The DICOM images were processed using 3D SolidWorks® (Dassault Systèmes, Waltham, MA, USA) to form 3-dimensional (3D) solid models of the proximal humerus. Separate 3D models of the cortical and trabecular bones were created using combinations of automatic threshold-based segmentation and visual distinctions. Under the supervision of two orthopedic surgeons (JG, AG), humeral head resections were simulated as would be done during TSA. All patients had provided written informed consent for the use of their images and data for research and publishing purposes.

**Fig. 1:**
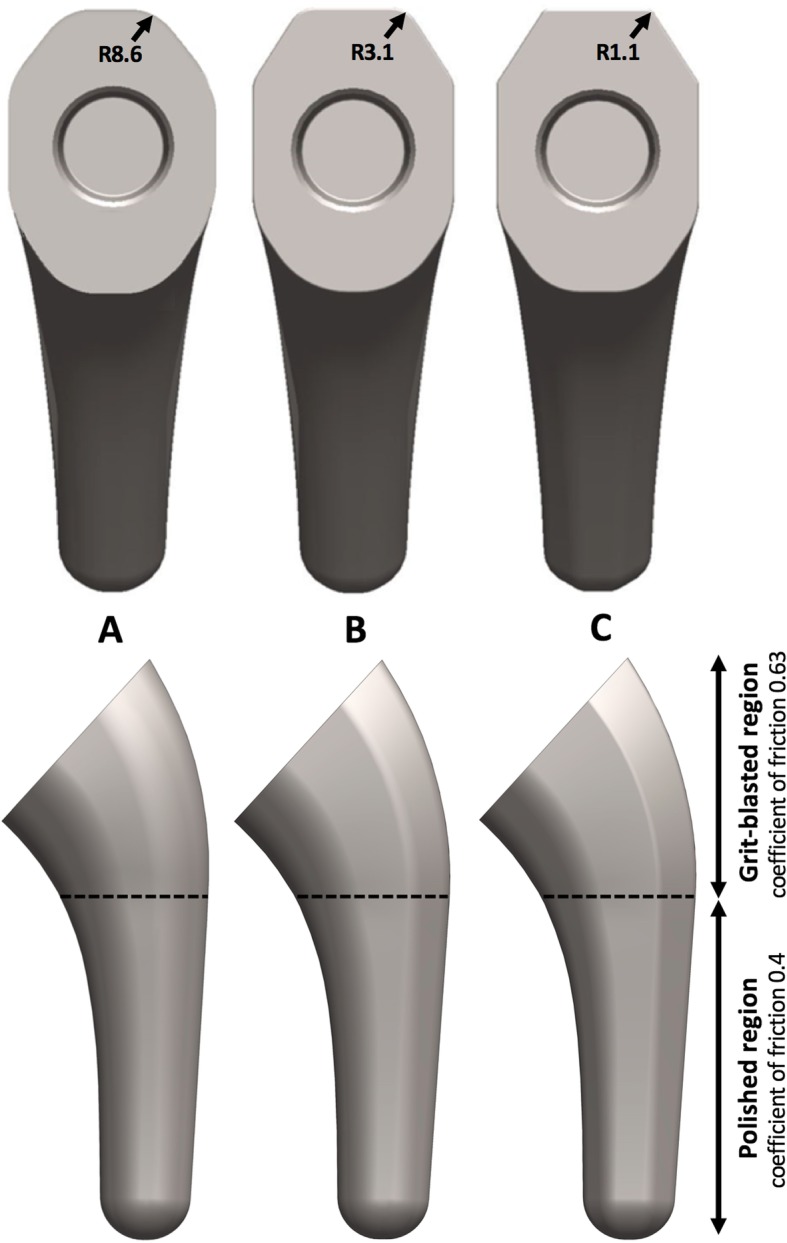
Three generic models of humeral stems presenting different proximal metaphyseal geometry: predominantly oval (**a**), semi-angular (**b**), and predominantly angular (**c**)

Computer models of 3 generic implants were created based on three design concepts: (A) predominantly oval (Ascend Flex, Wright-Tornier, Montbonnot, France), (B) semi-angular (ISA Onlay, Move-Up, Alixan, France), and (C) predominantly angular (Equinox, Exactech Inc., Gainesville, FL, USA) (Fig. 1). The stems were designed with the same neck-shaft angle (132.5°), head offset (6.2 mm), and humeral head geometry, but differed in the proximal metaphyseal area with different fillet radii of the supero-lateral edge: 8.6 mm (predominantly oval), 3.1 mm (semi-angular) and 1.1 mm (predominantly angular). The humeral heads were designed with a radius-to-height ratio of 1.00:0.76. The coefficient of friction assigned to the proximal grit-blasted metaphyseal region was 0.63, while that assigned to the cylindrical polished diaphyseal region was 0.4 (Grant et al., 2007; Kuiper & Huiskes, 1996; Razfar et al., 2016). Each humeral stem model was created in 9 sizes, increasing by increments of 2 mm in the antero-posterior (AP) direction and increments of 1 mm in the medio-lateral (ML) direction.

**Fig. 2:**
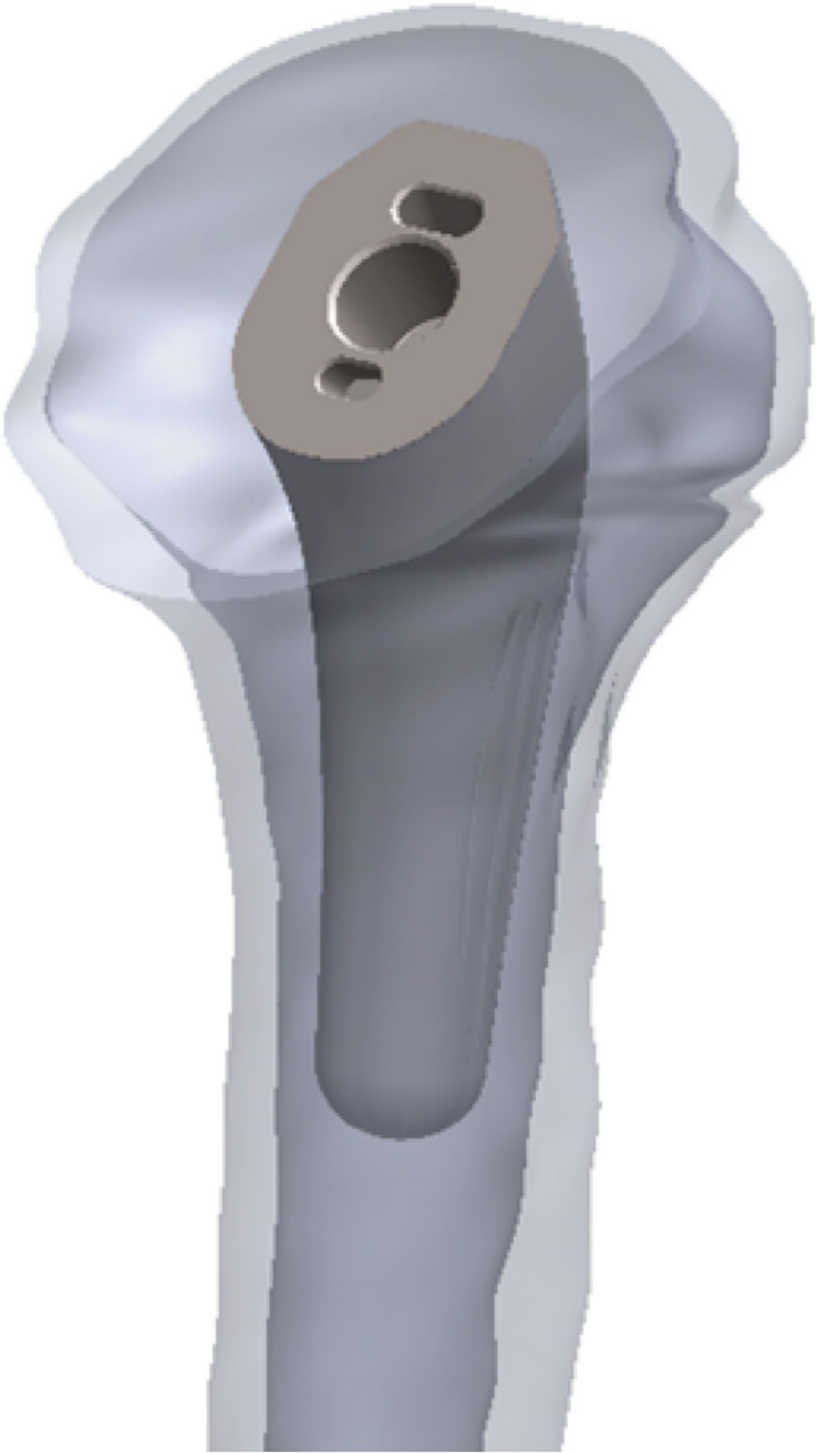
Implant positioning in the generated bone model

To re-create surgical placement in a repeatable manner across all models, anatomic landmarks were used as reference points, such that the native and prosthetic head centers have the same 3D coordinates, and the stem is aligned with the diaphyseal axis. Stem sizing was established by selecting the component that makes contact with the cortical diaphysis (oversized), one increment smaller (normosized), and two increments smaller (undersized). After implant positioning and sizing, all model components were transferred from SolidWorks to ADAMS (Adams, MSC Software Corporation, Santa Ana, CA) (Fig. 2). Bone was meshed using an average of 2 mm (maximum value) quadratic tetrahedral elements, based on mesh convergence analysis. The reliability of our model was confirmed using convergence testing. Careful mesh planning ensured that identical mesh parameters could be used for different implant geometries to allow comparisons of Von Mises stresses in different regions.

In agreement with previous studies, cortical bone was modeled as a homogeneous isotropic material with a Young’s modulus of 20 GPa and Poisson’s ratio 0.3 (Bayraktar et al., 2004; Rho et al., 1993). For trabecular bone, the Young’s modulus was applied on an element-by-element basis and calculated using corresponding CT densities, as described previously (Austman et al., 2009; Carter & Hayes, 1977; Leung et al., 2009; Morgan et al., 2003; Schileo et al., 2008; Taddei et al., 2006). The density-modulus relationship chosen for this study, which was reported by Morgan et al. (Morgan et al., 2003), is specific to trabecular bone:

$$ \mathrm{Equation}\ 1:\mathrm{E}=8920{\uprho}^{1.83} $$

Where *E* is Young’s modulus and ρ is the apparent bone density, calculated from Hounsfield Units.

All implant components were meshed using appropriately sized quadratic tetrahedral elements, and assigned properties of titanium (Ti6Al4V) (*E* = 115 GPa, ν = 0.31). To render our results comparable to the study of Razfar et al. (Razfar et al., 2016), the same force magnitude was applied, assuming a median mass of 88.3 kg for all patients, at a single abduction angle of 45°. The resultant force vector was therefore: Fx 160 N, Fy -440 N, Fz -210 N.

**Fig. 3:**
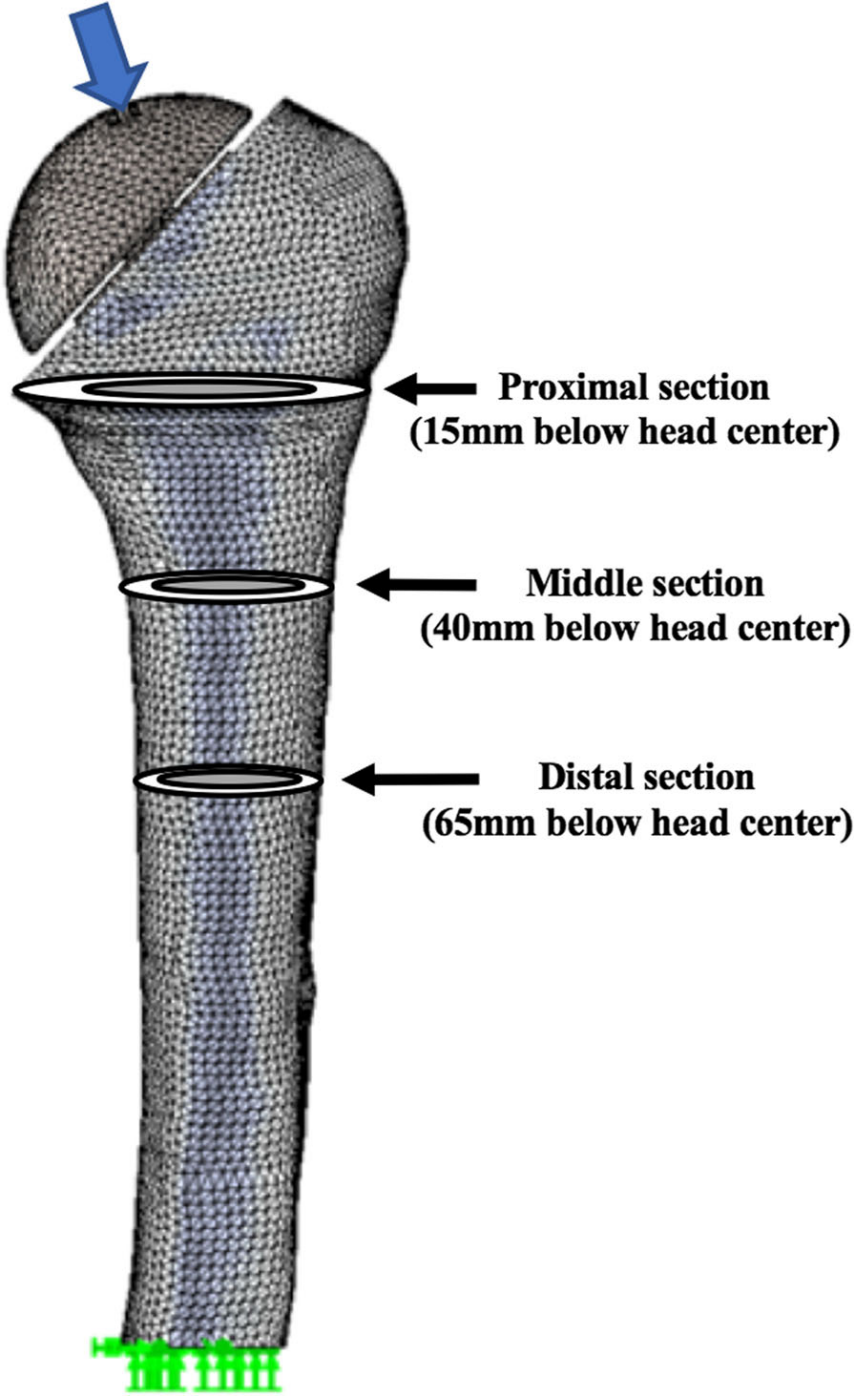
Meshed model of an implanted humerus, rigidly fixed at the distal part, indicating the direction of the proximal load towards the center of the prosthetic head

Finite element analyses (FEA) were performed with the humerus rigidly constrained at its distal end. To simulate the joint reaction force acting on the humerus, a load was directed from the joint surface toward the center of the humeral head, according to in vivo implant data (Fig. 3). A total of 20 analyses were performed using 4 FEA models (1 intact humerus + 3 implanted humeri) for each of the 5 subjects. To quantify changes in the proximal humerus after TSA, mean bone stresses were compared for the implanted versus intact humerus at 3 transverse sections: proximal (15 mm below head center), middle (40 mm below head center), and distal (65 mm below head center). Torsional stability was assessed by measuring the maximum angular displacement of the stem, defined as the angle (degrees) through which the stem moves around the diaphyseal axis.

## Results

### Trabecular bone stresses

**Fig. 4:**
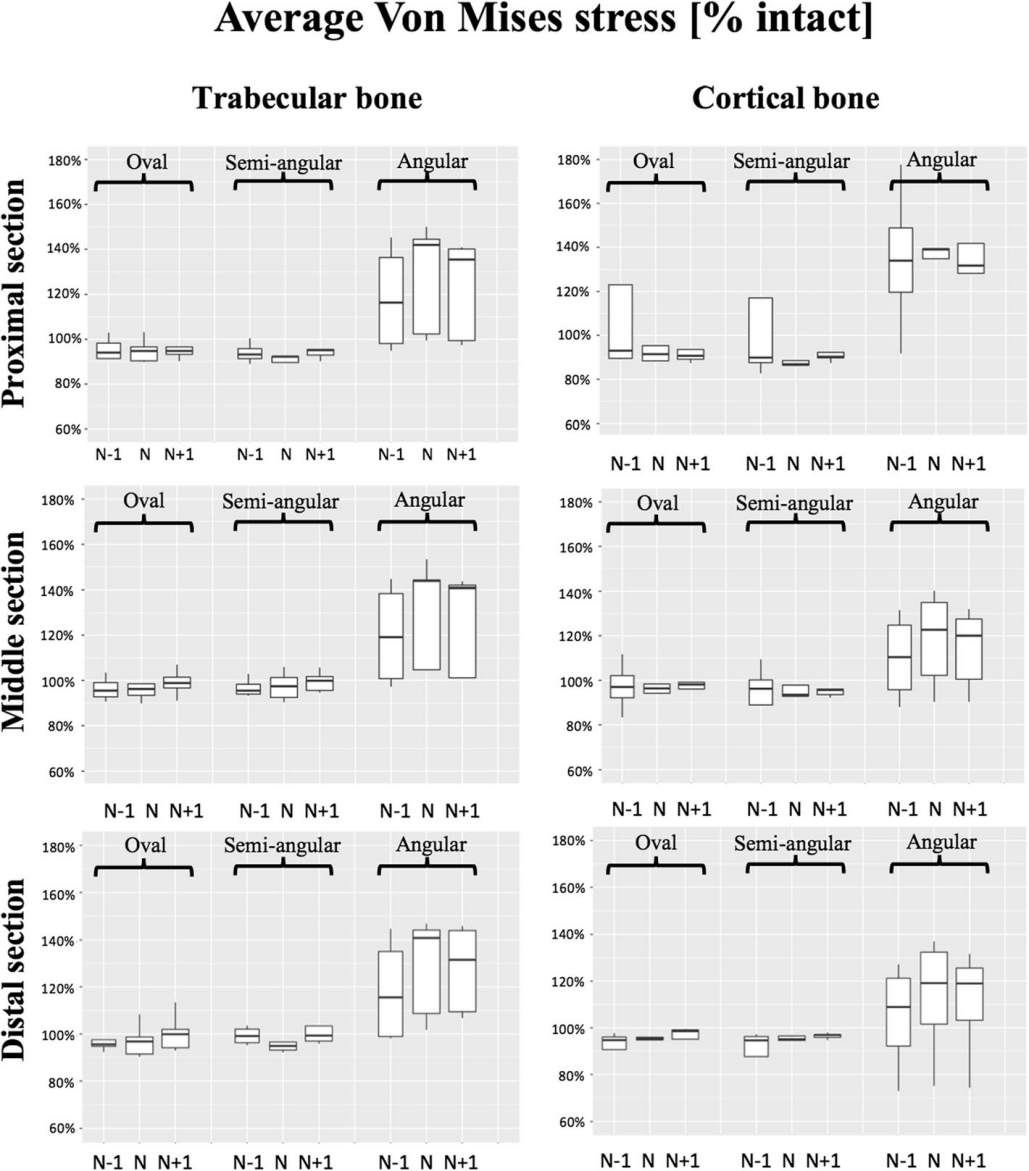
Average Von Mises stress in trabecular and cortical bone at proximal, middle and distal sections. Average stresses are presented as a percentage of the intact bone stress in each section

Bone stress changes within trabecular bone followed the same pattern within all sections, and were mainly associated with implant design rather than implant size (Fig. 4). Considering the normosized stems, the angular design *increased* the physiologic stresses at the proximal section by a median of 42%, while the oval and semi-angular designs *reduced* them by a median of 5% and 8%, respectively.

### Cortical bone stresses

Bone stress changes within cortical bone followed the same pattern within all sections, and were also mainly associated with implant design rather than implant size (Fig. 4). Considering the normosized stems, the angular design *increased* the physiologic stresses at the proximal section by a median of 39%, while the oval and semi-angular designs *reduced* them by a median of 9% and 13%, respectively.

### Torsional stability

**Fig. 5:**
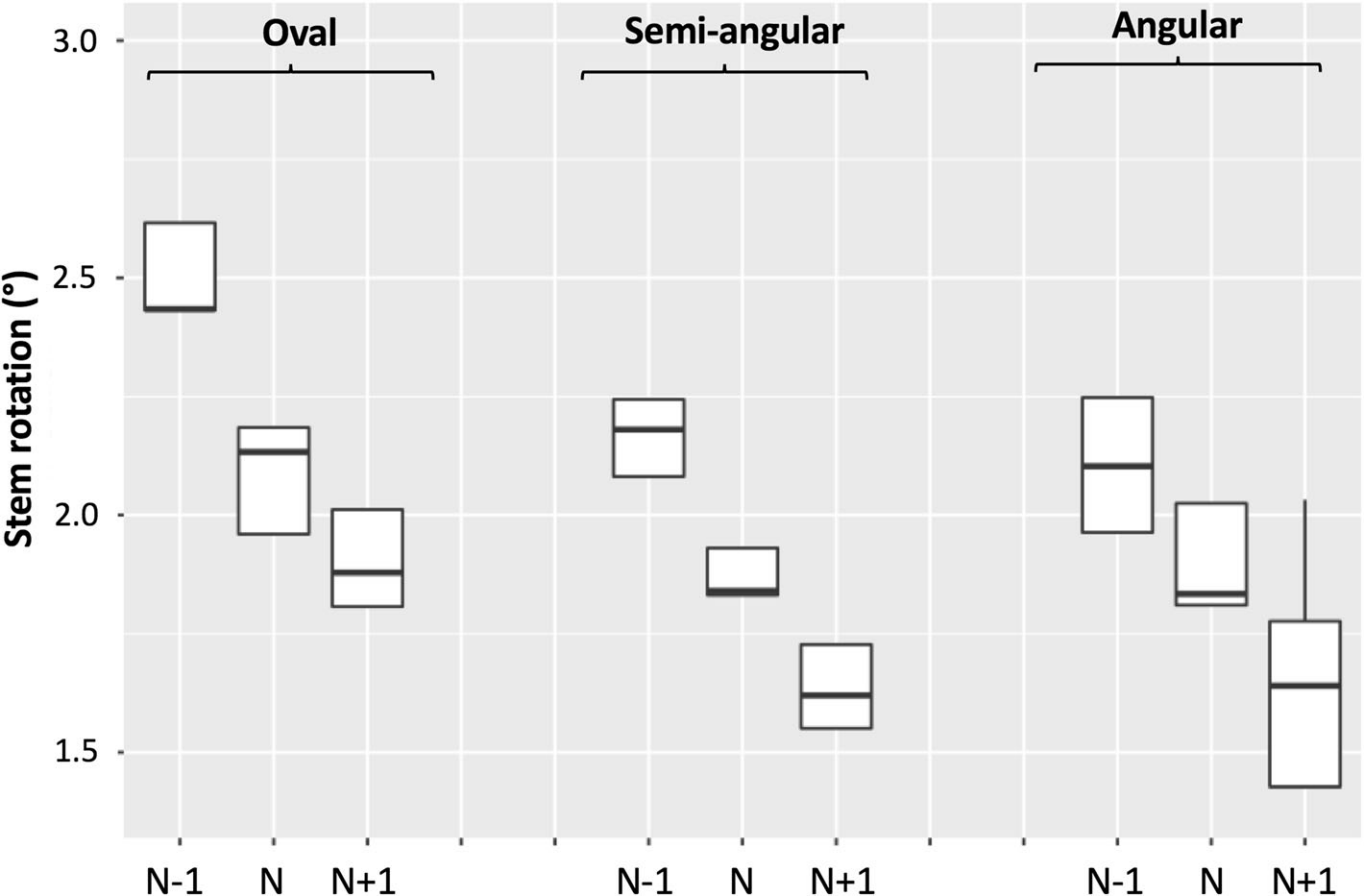
Torsional stability

Stem torsional stability was associated with both implant design and size (Fig. 5). Considering the normosized stems, the oval design exhibited a median rotation of 2.1°, while the semi-angular and angular designs exhibited median rotations of 1.8°. The rotation of the oversized oval design (1.9°) was comparable to the rotations of the semi-angular (1.8°) and angular designs (1.8°).

## Discussion

The principal finding of this study was that the semi-angular stem design granted an adequate compromise between physiologic stress distributed by the oval stem design and torsional stability of the angular stem design. Several authors evaluated the mechanical behavior of glenoid implants within the scapula (Buchler & Farron, 2004; Chevalier et al., 2016; Iannotti et al., 2005; Pomwenger et al., 2014; Stone et al., 1999), but, very few studied the stress distribution around humeral (Quental et al., 2012; Razfar et al., 2016). This study is the first to investigate the influence of humeral stem size and proximal stem geometry on stress distributions and torsional stability within periprosthetic bone.

Joint reconstruction aims to reproduce native patient anatomy and physiologic stress distributions. Razfar et al. (2016) demonstrated that short stems transfer loads more proximally and thereby reduce stress-shielding observed with long stems are implanted. It is worth noting, however, that there is very little long-term clinical and radiographic data reported for short stems (Denard et al., 2018) and it may not be appropriate to consider the various available designs as a single group (Yan et al., 2017). Several authors reported satisfactory short-term outcomes for oval stems (Morwood et al., 2017; Schnetzke et al., 2017; Szerlip et al., 2018) and equally good outcomes for angular stems with low incidences of radiolucent lines and radiographic loosening (Gilot et al., 2015; King et al., 2015).

Our study demonstrated that the oval and semi-angular stem designs reproduced native bone stresses while the angular stem design exacerbated them by 39%–42%. Such considerable stress changes could lead to periprosthetic cracks or fatigue fractures which can be difficult to manage (Keener et al., 2017; Quental et al., 2012; Verborgt et al., 2007). Our study also revealed that the angular and semi-angular stem designs had better torsional stability compared to the oval stem design. Therefore, the semi-angular stem has the benefits of granting the physiologic stress distribution of the oval stem design and the adequate torsional stability of the angular stem design.

While surgeons tend to implant oversized stems to improve primary fixation, numerous studies demonstrated that increasing humeral stem diameter could cause stress-shielding, and potentially lead to peripheral bone resorption (Nagels et al., 2003; Razfar et al., 2016). Likewise, Denard et al. (2018) supported that implanting a larger stem leads to more distal fixation and subsequent under-loading within the proximal humerus. Furthermore, Inoue et al. (Inoue et al., 2017) found that a high “occupation ratio” (canal fill ratio) increases risks of bone resorption. For these reasons, some authors recommend to implant the stem of smallest width that achieves adequate stability (Denard et al., 2018), but care should be taken to avoid excessive stem undersizing, which could lead to loosening, subsidence or misalignment (Goetzmann et al., 2017; Raiss et al., 2018). Our study revealed that the torsional stability of the normosized semi-angular stem is comparable to that of the oversized oval stem. The semi-angular stem design could therefore help surgeons achieve adequate stability and physiologic proximal stresses, without oversizing their stems.

The limitations of this study were (i) the non-validation of the FEA models by in-vivo cadaver tests to ensure that estimated bone stresses correspond to true physiologic stresses, (ii) the choice of a constant joint reaction force value which is supposed to be patient specific, and (iii) the use of cortical contact to define stem size rather than filling ratios that were described in recent studies (Raiss et al., 2018; Schnetzke et al., 2018). The main strengths of this study are the number and anatomic variability of humeri selected, as well as the use of CT scans of patients who later underwent TSA which represents our population of interest.

## Conclusion

The present study revealed that the semi-angular stem design granted an adequate compromise between physiologic stress distributed by the oval stem design and torsional stability of the angular stem design. Surgeons should be aware of the various benefits and drawbacks of the different humeral stem designs and adjust their implant sizing and positioning accordingly, to ensure adequate torsional stability and physiologic stress transfer.

## Abbreviations

3D: 3-Dimensional
CT: Computed Tomography
FEA: Computed Tomography
ML: Medio-Lateral
TSA: Total Shoulder Arthroplasty
